# Deletion of *DP148R*, *DP71L*, and *DP96R* Attenuates African Swine Fever Virus, and the Mutant Strain Confers Complete Protection against Homologous Challenges in Pigs

**DOI:** 10.1128/jvi.00247-23

**Published:** 2023-04-05

**Authors:** Xiaolan Qi, Tao Feng, Zhao Ma, Linlin Zheng, Huanan Liu, Zhengwang Shi, Chaochao Shen, Pan Li, Panxue Wu, Yi Ru, Dan Li, Zixiang Zhu, Hong Tian, Sen Wu, Haixue Zheng

**Affiliations:** a State Key Laboratory for Animal Disease Control and Prevention, College of Veterinary Medicine, Lanzhou University, Lanzhou Veterinary Research Institute, Chinese Academy of Agricultural Sciences, Lanzhou, China; b State Key Laboratory of Agrobiotechnology, College of Biological Sciences, China Agricultural University, Beijing, China; Lerner Research Institute, Cleveland Clinic

**Keywords:** ASFV, *DP148R*, *NL*, *UK*, live attenuated vaccine

## Abstract

The African swine fever virus (ASFV) has caused a devastating pandemic in domestic and wild swine, causing economic losses to the global swine industry. Recombinant live attenuated vaccines are an attractive option for ASFV treatment. However, safe and effective vaccines against ASFV are still scarce, and more high-quality experimental vaccine strains need to be developed. In this study, we revealed that deletion of the ASFV genes *DP148R*, *DP71L*, and *DP96R* from the highly virulent isolate ASFV CN/GS/2018 (ASFV-GS) substantially attenuated virulence in swine. Pigs infected with 10^4^ 50% hemadsorbing doses of the virus with these gene deletions remained healthy during the 19-day observation period. No ASFV infection was detected in contact pigs under the experimental conditions. Importantly, the inoculated pigs were protected against homologous challenges. Additionally, RNA sequence analysis showed that deletion of these viral genes induced significant upregulation of the host histone H3.1 gene (*H3.1*) and downregulation of the ASFV *MGF110-7L* gene. Knocking down the expression of *H3.1* resulted in high levels of ASFV replication in primary porcine macrophages *in vitro*. These findings indicate that the deletion mutant virus ASFV-GS-Δ18R/NL/UK is a novel potential live attenuated vaccine candidate and one of the few experimental vaccine strains reported to induce full protection against the highly virulent ASFV-GS virus strain.

**IMPORTANCE** Ongoing outbreaks of African swine fever (ASF) have considerably damaged the pig industry in affected countries. Thus, a safe and effective vaccine is important to control African swine fever spread. Here, an ASFV strain with three gene deletions was developed by knocking out the viral genes *DP148R* (*MGF360-18R*), *NL* (*DP71L*), and *UK* (*DP96R*). The results showed that the recombinant virus was completely attenuated in pigs and provided strong protection against parental virus challenge. Additionally, no viral genomes were detected in the sera of pigs housed with animals infected with the deletion mutant. Furthermore, transcriptome sequencing (RNA-seq) analysis revealed significant upregulation of histone H3.1 in virus-infected macrophage cultures and downregulation of the ASFV *MGF110-7L* gene after viral *DP148R*, *UK*, and *NL* deletion. Our study provides a valuable live attenuated vaccine candidate and potential gene targets for developing strategies for anti-ASFV treatment.

## INTRODUCTION

African swine fever (ASF) is a viral disease with a mortality rate of up to 100%. It was first reported in Kenya in 1921 and has continued to spread in Africa and Eurasia, with an outbreak in the Caucasus region in 2007 and a recent outbreak in East Asia (1), which produces more than 50% of the global supply of pork (2). Currently, ASF is endemic to more than 40 countries in Africa, Europe, and Asia (3). The causative pathogen, African swine fever virus (ASFV), is the only member of the genus *Asfivirus* within the family *Asfarviridae* that contains a double-stranded DNA genome of 170 to 190 kb. Its genome consists of 167 genes, and the functions of many viral genes remain unknown (4, 5). The ASF epidemic has seriously hampered the development of pig breeding and the economy in the affected areas. Current control strategies mainly depend on restricting the movement of animals, culling infected pigs, and implementing methods of biosafety control (6, 7).

Developing a safe and efficient vaccine against ASFV is of great importance for the global control of the ASF epidemic. Experimental vaccines based on live-attenuated ASFV instead of inactivated viruses induce strong protection against homologous (8, 9) and occasionally heterologous challenges (10, 11). To date, experimental vaccines produced via the deletion of multiple genes, including *TK* (*A240L*), *CD2v* (*EP402R*), *9GL* (*B119L*), *UK*, *DP148R*, *I177L*, and *MGF360/505*, individually or in combination, have been proven to be effective (8–10, 12–14). Deletion of *DP148R* causes different effects on the genotype I isolate Benin 97/1 and the genotype II isolate Georgia 2007/1. BeninΔDP148R is remarkably attenuated *in vivo* and provides a high level of protection against homology challenges (13), whereas the virulence of GeorgiaΔDP148R is not diminished (15). The nonessential ASFV genes *NL* and *UK* are important virulence determinants in pigs (12, 16). A previous study revealed that deletion of the viral *NL* gene attenuates the E70 ASFV strain (17). The ASFV gene *UK* has also been proven to be an important virulence gene for ASFV strains E70 (18), Georgia 2007 (12), and SY18 (19). Notably, strains obtained via the deletion of *NL* and *UK* from OUT T88/3 (a naturally attenuated virus strain) and the Georgia isolate failed to induce protection against homologous challenge (20, 21).

In 2022, the Vietnamese government approved an ASFV vaccine named NAVET-ASFVAC, the first commercially available vaccine for ASFV worldwide. It is a live attenuated vaccine (LAV), and the vaccine strain was produced by deleting the *I177L* gene from the highly virulent ASFV isolate Georgia 2007/1 (ASFV-G) (8, 22, 23). As LAVs with gene deletions hold great promise for developing ASFV vaccines, more effective and safer ASFV vaccine candidates must be developed.

These three virulence-related genes, *DP148R*, *NL*, and *UK*, have different effects on virulence attenuation and immune protection in different strains. To evaluate the effect of combined deletion of these three genes on the now-prevalent strains in the field, in this study, we used the ASFV strain CN/GS/2018 (ASFV-GS) as the original strain to generate and evaluate a triple-gene-deletion mutant, ASFV-GS-Δ18R/NL/UK. The ASFV-GS isolate was isolated from a field in Gansu Province, China (24, 25). Based on nucleotide sequence analysis, this strain was derived from the highly virulent ASFV-G isolate (sequence homology > 99%), which belongs to genotype II. We found that this deletion mutant virus strain was highly attenuated in pigs, providing complete protection against the ASFV-GS challenge. On the other hand, genomes of ASFV encode more than 150 proteins, and there must be interactions between these viral proteins. Following the deletion of a viral gene(s), the expression of the remaining genes may be affected. Here, we further analyzed the expression levels of host genes and ASFV genes via transcriptome sequencing (RNA-seq) of bone marrow-derived macrophage (BMDM) cultures infected with either ASFV-GS-Δ18R/NL/UK or its parental strain. Significant downregulation of the viral *MGF110-7L* gene was observed in the deletion mutant virus strain. Our study provides targets for the development of LAVs and antiviral treatments.

## RESULTS

### Production of the deletion mutant ASFV-GS-Δ18R/NL/UK.

**FIG 1:**
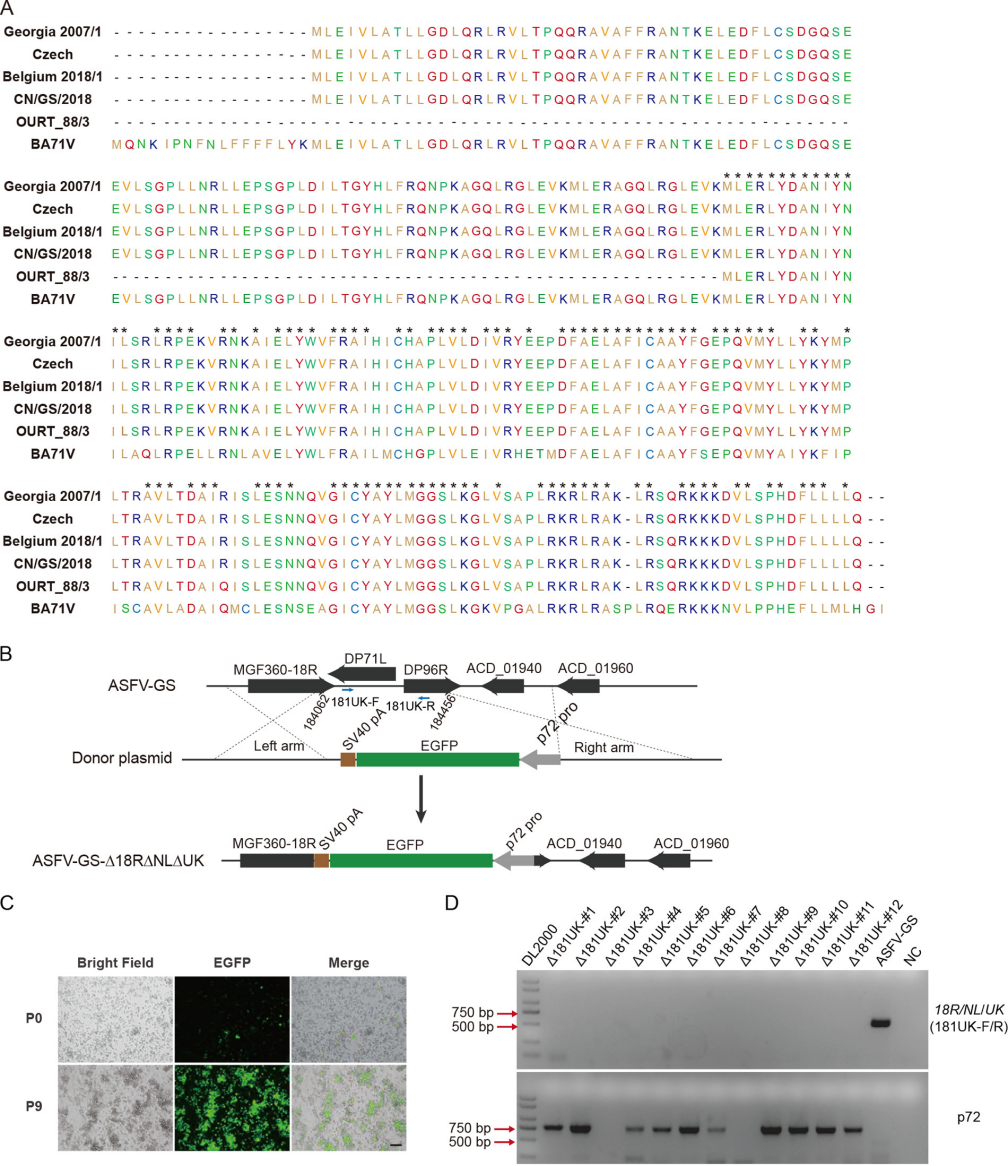
Generation of the deletion mutant ASFV-GS-Δ18R/NL/UK. (A) Comparisons of ASFV DP148R protein sequences among indicated ASFV isolates. Asterisks indicate the amino acid residues that were consistent among all virus strains. (B) Schematic of the generation of the deletion mutant via homologous recombination. *DP148R*, *NL*, and *UK* genes were replaced with the p72-EGFP cassette. Two blue arrows highlighting 181UK-F and 181UK-R indicate the sites of primers for purity analysis. (C) A low number of EGFP-positive cells was detected in BMDMs at the beginning of purification, and nearly all BMDM cultures emitted EGFP signals at the end of purification. (D) PCR results of the deletion mutant purity. Deionized water was used as the negative control (NC). The ASFV-GS genome was used as the positive control. Δ181UK-#1 to -#12 represent different deletion mutant strains. The viral gene *B646* (p72) was used as the reference gene.

The *DP148R*, *NL*, and *UK* genes of ASFV-GS are located on DNA strands at positions 183372 to 184085, 184068 to 184280, and 184379 to 184669, respectively. ASFV genotype I (OUR T 88/3 and BA71V) and genotype II (Georgia 2007/1, Czech, Belgium 2018/1, and CN/GS/2018) viral proteins were evaluated for sequence similarity because they are most prevalent in the Russian Federation, the People’s Republic of China, and Southeast Asia (26, 27). Results showed that the protein sequence homology of DP148R was as high as 98.8% among the four genotype II strains, compared to only 39.5% between genotype I and genotype II virus strains (Fig. 1A). Meanwhile, for the two genotype I isolates and the four ASFV genotype II isolates, there was no variation in the homology of the NL protein sequence. The genotype I and II strains were found to differ in five residues, although generally there was a high degree of amino acid sequence similarity (92.75%) between them (see Fig. S1A in the supplemental material). ASFV genotypes I and II show no variations in the homology of UK protein sequences. The genotype I and II strains were found to differ in seven residues. There was 92.63% homology in the UK protein sequence between the two genotypes (Fig. S1B). According to these findings, the proteins NL and UK displayed substantial sequence homology among genotype II viral strains but minimal sequence homology between genotypes I and II.

To construct the deletion mutant, genomic segments covering *DP148R*, *NL*, and *UK* were replaced with the reporter gene cassette p72-GFP via homologous recombination (Fig. 1B). Thus, these three genes were assumed to be functionally lost. The deletion mutant was purified using BMDMs. At the beginning of purification (P0), only a few green fluorescent protein (GFP)-positive cells were detected. After successive cycles of selecting cells, freezing them, and thawing them, the percentage of GFP-positive cells reached approximately 100% (Fig. 1C). PCR was performed to check the purity of the deletion mutant virus stock, and the results suggested no contamination with the wild-type virus (Fig. 1D), indicating that the deletion mutant (ASFV-GS-Δ18R/NL/UK) was successfully constructed.

### Deletion of the targeted genes did not affect the expression of their neighboring genes or ASFV growth kinetics *in vitro*.

**FIG 2:**
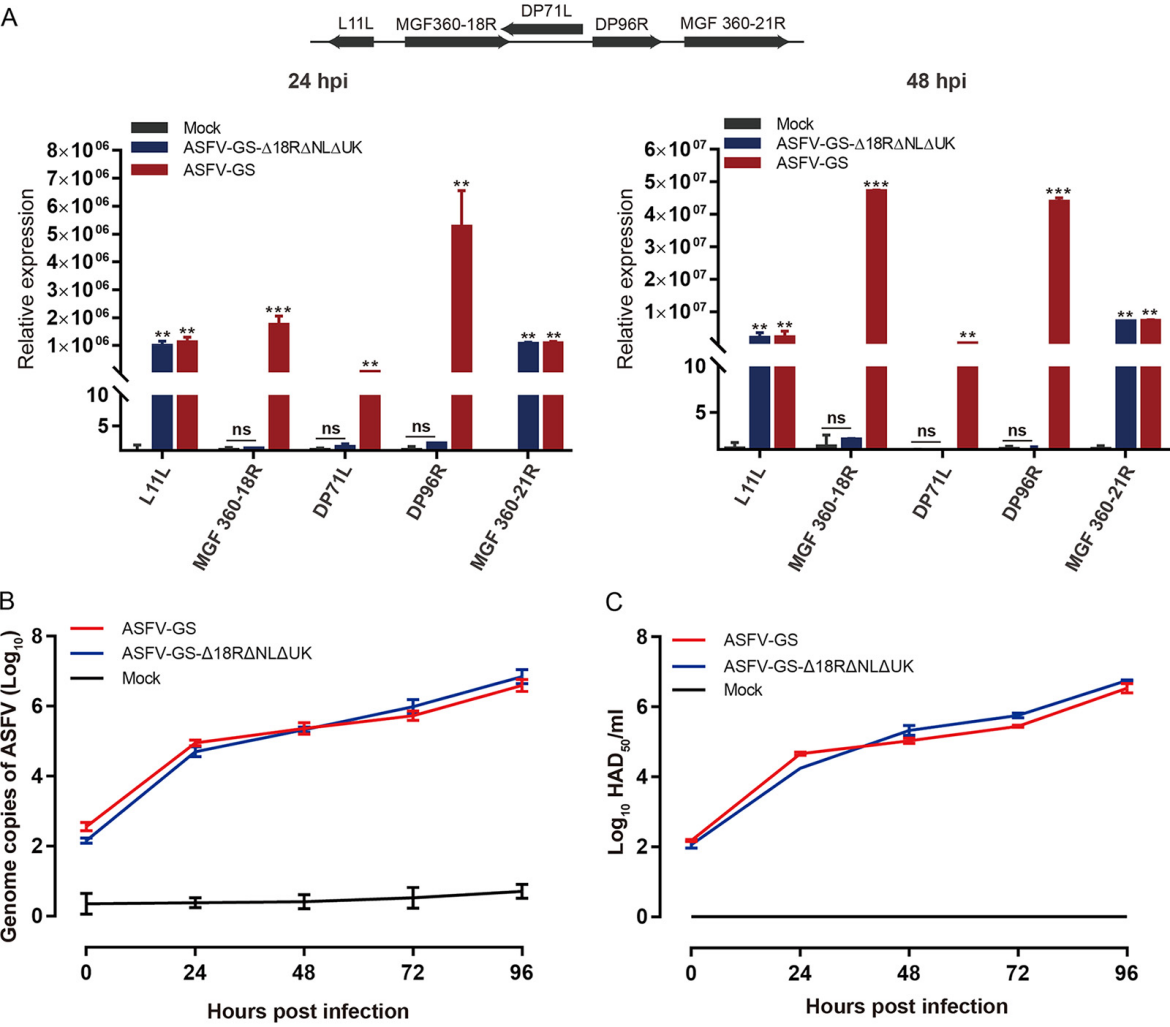
Characterization of ASFV-GS-Δ18R/NL/UK. (A) Relative expression analysis of targeted genes and neighboring genes of the deletion mutant against ASFV p72. The values are means and standard deviations (SD). **, *P* < 0.01; ***, *P* < 0.001; ns, not significant. (B and C). Growth kinetics of ASFV-GS and ASFV-GS-Δ18R/NL/UK *in vitro*. Pig BMDMs were infected with the indicated virus strain, and viruses were harvested at the indicated time points. The collected samples were assessed using qPCR (B) and a hemagglutination assay (C). Samples mock infected with ASFV were used as negative controls (Mock).

To test the expression levels of the target genes, quantitative PCR (qPCR) was performed on BMDM cultures infected with ASFV-GS or the deletion mutant. At both 24 h postinfection (hpi) and 48 hpi, transcription of these three genes was not detected in the deletion mutant group, unlike in the ASFV-GS group (Fig. 2A). These results further confirmed the functional loss of targeted genes. To determine whether gene deletion affects neighboring genes, we examined the expression of *L11L* and *MGF360-21R*, located upstream and downstream of the deleted genes. No significant differences were observed between the mutant and wild-type virus groups (Fig. 2C).

A one-step growth curve between the deletion mutant and its parental strain was examined to test the growth kinetics of ASFV-GS-Δ18R/NL/UK *in vitro*. ASFV genome copy number was determined using qPCR; the results indicated no significant differences between the capacity of the parental strain ASFV-GS and that of the recombinant virus to replicate *in vitro* (Fig. 2B). Furthermore, the 50% hemadsorbing dose (HAD_50_) test did not reveal any discernible differences (Fig. 2C). These results imply that deletion of the viral genes *DP148R*, *NL*, and *UK* had no significant impact on ASFV replication in BMDMs. These data suggest that functional deletion of the *DP148R*, *NL*, and *UK* genes has no apparent effect on the expression of their neighboring genes or on ASFV growth kinetics *in vitro*.

### The deletion mutant is highly attenuated in pigs.

To investigate the virulence of ASFV-GS-Δ18R/NL/UK, five pigs (70 to 80 kg) were intramuscularly injected with 10^4^ HAD_50_ of ASFV-GS-Δ18R/NL/UK and observed for 19 days postinoculation (dpi). Three contact pigs were mock infected with cell culture medium and cohabitated with the inoculated pigs to detect virus shedding from infected animals. The control group consisted of five pigs that were injected intramuscularly (i.m.) with 10^4^ HAD_50_ of ASFV-GS.

**FIG 3:**
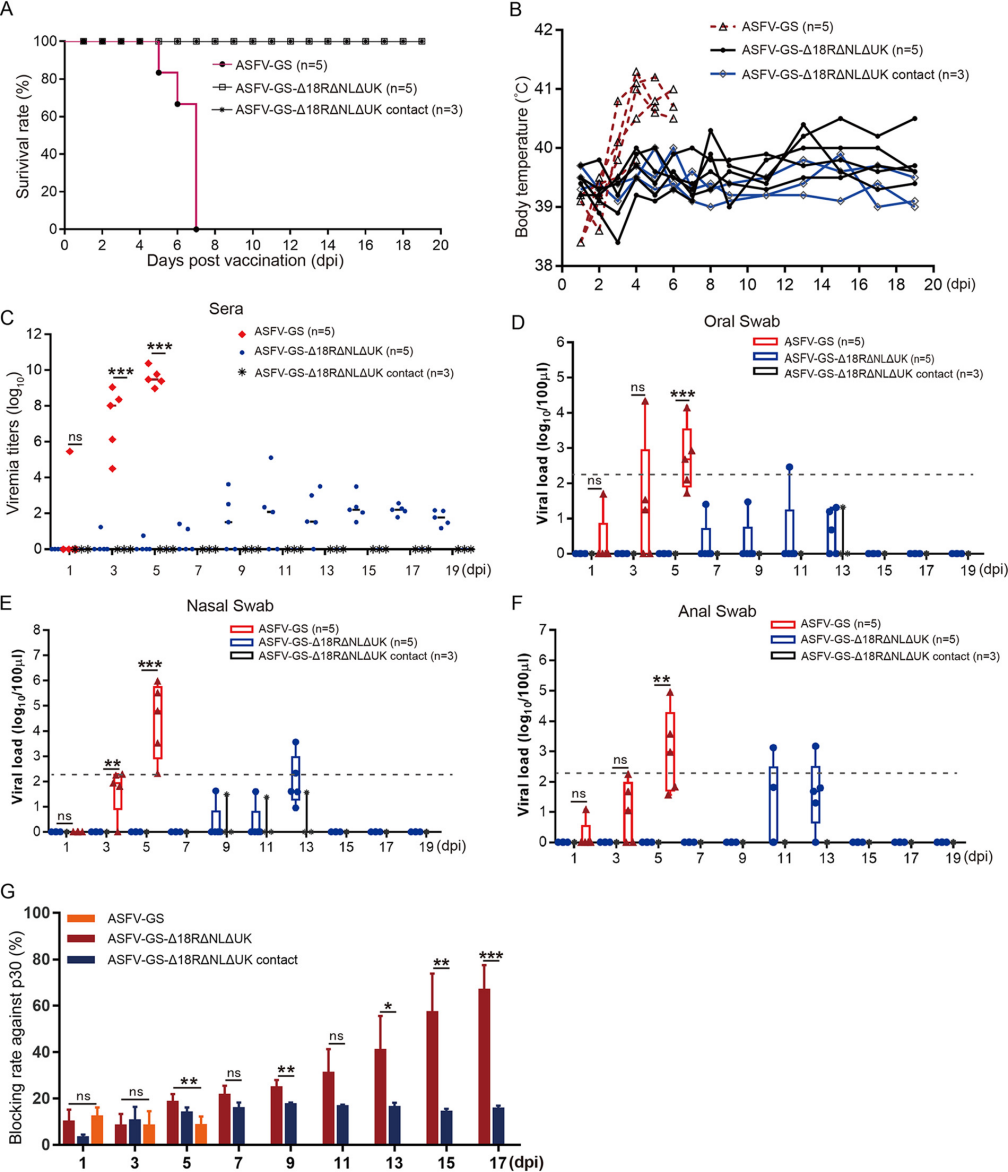
Virulence assay of the deletion mutant in pigs. (A) Survival rates of pigs inoculated with ASFV-GS and ASFV-GS-Δ18R/NL/UK. In the contact group, pigs were mock-infected and housed with the pigs inoculated with ASFV-GS-Δ18R/NL/UK. (B) Body temperatures of pigs intramuscularly inoculated with ASFV-GS, ASFV-GS-Δ18R/NL/UK, or cell culture medium. Each curve represents values for one animal. (C) Viral load detection in the sera of pigs inoculated with the indicated virus strains or cell culture medium. (D) Viral load from the oral swabs of pigs inoculated with ASFV-GS, ASFV-GS-Δ18R/NL/UK, or cell culture medium. (E) Viral load from the nasal swabs of pigs inoculated with ASFV-GS, ASFV-GS-Δ18R/NL/UK, or cell culture medium. (F) Viral load from anal swabs of pigs inoculated with ASFV-GS, ASFV-GS-Δ18R/NL/UK, or cell culture medium. (G) p30-specific antibody profiles of pigs infected with the indicated virus strains or cell culture medium. The values are means and SD. *, *P* < 0.05; **, *P* < 0.01; ***, *P* < 0.001; ns, not significant. Different-colored symbols indicate different groups. Dashed lines indicate the lower limit of the qPCR method.

Survival rate and body temperature were calculated at the end of the experiment. As shown in Fig. 3A and B, all pigs in the control group died before 7 dpi, body temperature began to increase at 3 dpi, and a high temperature (>40°C) was maintained until the pigs died. The pigs inoculated with ASFV-GS-Δ18R/NL/UK and mock infected survived the observation period. These pigs did not develop fever, except one which showed a slight increase in body temperature at the end of the observation period. Serum samples from all animals were collected every other day during the experiment, and the viral load in the sera was determined. Notably, the viral load in the sera of the control pigs was as high as 10^8^ HAD_50_ of ASFV per mL, whereas that in the sera of the inoculation group remained stable at less than 10^3^ HAD_50_ per mL (Fig. 3C). This observation suggests that ASFV infection did not occur in naive pigs housed with inoculated animals under the experimental conditions. Furthermore, we tested the viral genomes in oral, nasal, and anal swabs collected from pigs of different groups. High-level viral loads were detected in ASFV-GS-inoculated pigs, low viral loads in ASFV-GS-Δ18R/NL/UK-inoculated pigs, and almost no virus in contact pigs (Fig. 3D to F). These findings suggest that the deletion mutant was attenuated and unable to infect contact pigs under experimental conditions.

### The deletion mutant provides strong protection against parental virus challenge.

**FIG 4:**
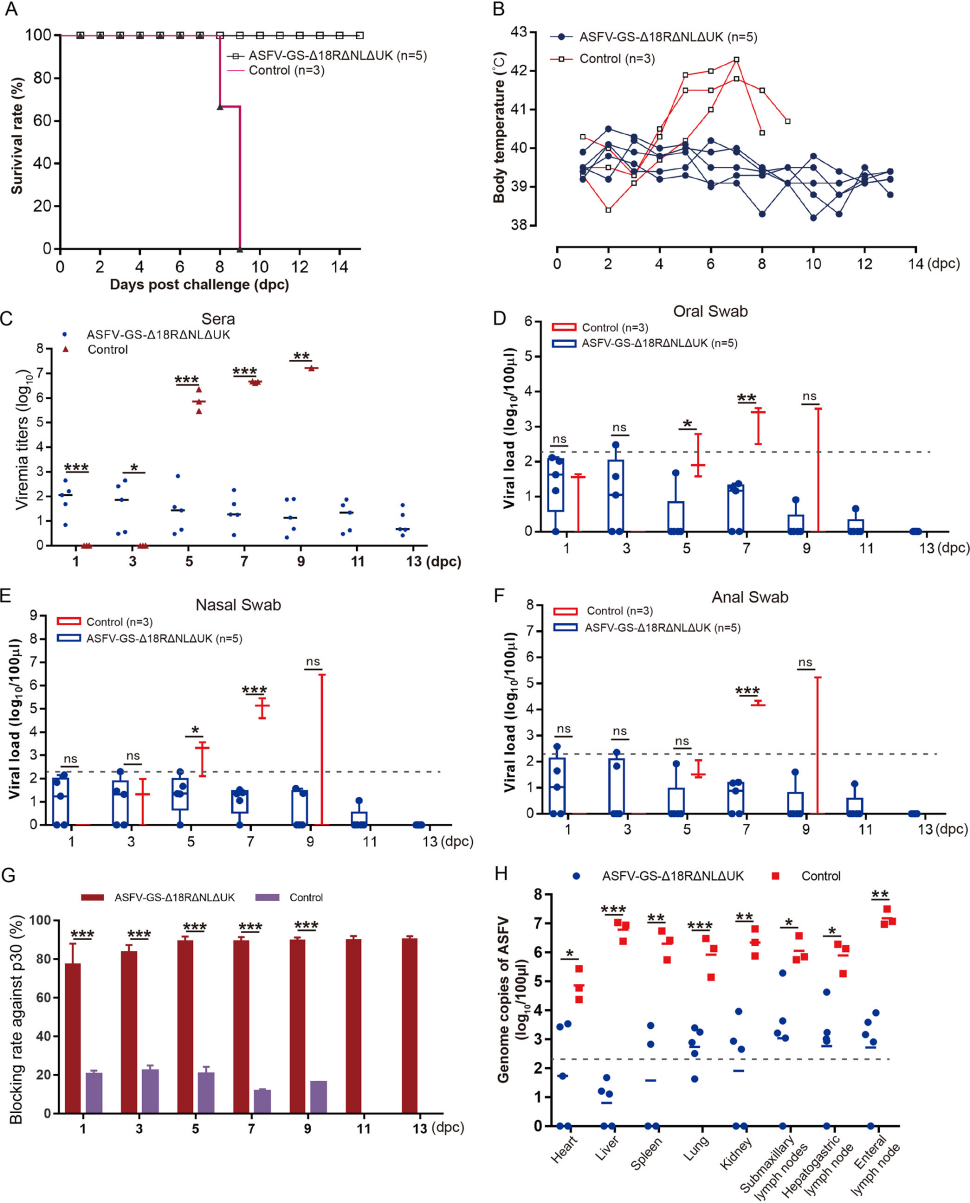
Protection assessment of the deletion mutant against homologous challenge. (A to C) Survival rate (A), body temperatures (B), and viral loads in the sera (C) of inoculated (ASFV-GS-Δ18R/NL/UK) or mock-inoculated (Control) pigs challenged with ASFV-GS. (D) Viral load test of oral swabs collected from challenged pigs inoculated or mock inoculated at the indicated time points. (E) Viral load assay of anal swabs collected from challenged pigs that had been inoculated or mock-inoculated. (F) Viral load test of anal swabs collected from challenged pigs, inoculated or mock-inoculated. (G) p30-specific antibody response in inoculated and mock-inoculated pigs. (H) Viral genome detection in tissues of pigs that had been inoculated and mock-inoculated before challenging with ASFV-GS. The values are means and SD. *, *P* < 0.05; **, *P* < 0.01; ***, *P* < 0.001; ns, not significant. Different-colored symbols indicate different groups. Dashed lines indicate the lower limit of the qPCR method.

To analyze the protective efficiency of the gene-deleted ASFV-GS-Δ18R/NL/UK virus strain, 19 days after intramuscular immunization with the deletion mutant, five pigs were challenged with 10^2^ HAD_50_ of the parental ASFV-GS viral strain by intramuscular injection. Three mock-inoculated pigs were challenged with the same dose of the parental virus strain using the same method of administration, and they served as controls. The survival rates and body temperatures are shown in Fig. 4A and B. All mock-inoculated pigs died within 9 days postchallenge (dpc) and developed ASFV-related fever. All five pigs in the inoculated group survived the challenge, and no classic ASFV-related symptoms were observed during the observation period. The viral load in the sera of these pigs was determined, and the results are shown in Fig. 4C. Viral replication was undetectable in the sera of control pigs at 3 dpc, and high viral loads started to emerge at 5 dpc (~10^6^ HAD_50_ per mL). In the inoculated group, viral loads were 10,000 times lower than those in the control group, and a low viral load was sustained throughout the observation period. Oral, nasal, and anal swabs were collected from pigs in both groups. The ASFV genome was detected in samples from both control and inoculated animals; the viral load of animals from the latter group was generally lower than that in the former group (Fig. 4D to F).

Antibody-mediated immune protection induced by ASFV proteins is not well understood (27). An optimized enzyme-linked immunosorbent assay (ELISA) kit was used to examine the ASFV p30 antibody response in experimental animals. The results showed that inoculated pigs developed a strong p30-specific response at 1 dpc, which remained high until the end of the observation period. However, the control pigs failed to respond to ASFV p30 infection until their death (Fig. 4G). At the end of the observation period, tissues such as the heart, liver, spleen, lungs, kidney, submaxillary lymph nodes, hepatogastric lymph nodes, and enteral lymph nodes were collected from three mock-inoculated and five inoculated pigs for viral DNA detection. The viral load in the control group pig samples was as high as 10^7^ genomic copies of ASFV per mL, whereas that in inoculated group pig samples was approximately 1,000-fold lower (Fig. 4H). These results indicate that the deletion mutant protects against homologous virus challenge.

### Increased replication of ASFV induced by the downregulation of the histone gene *H3.1*.

**FIG 5:**
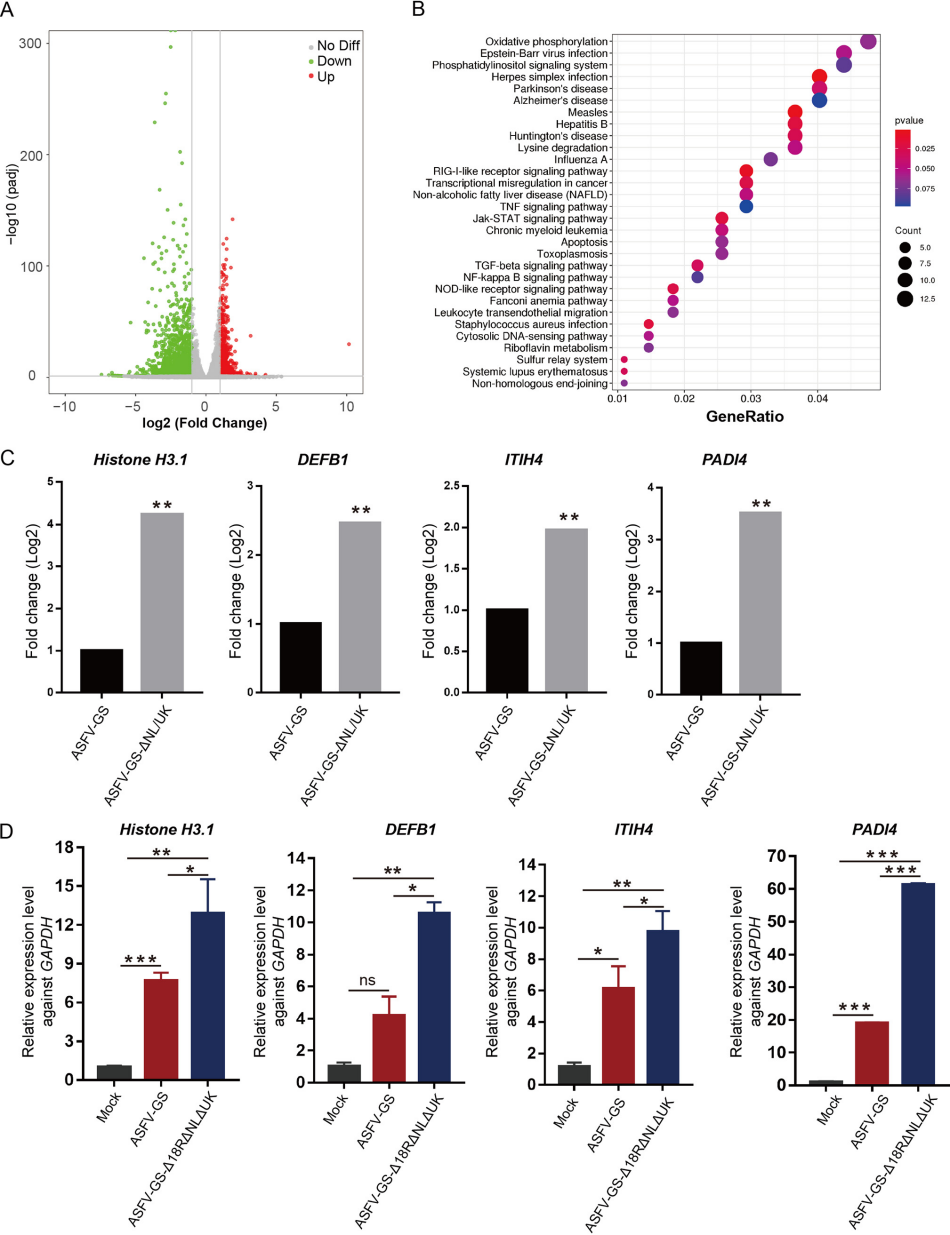
RNA sequencing analysis of the transcriptome of BMDMs infected with different virus strains. (A) Volcano plot showing DEGs. Green and red plots indicate downregulated and upregulated genes, respectively. (B) KEGG analysis of the DEGs. (C) Gene expression levels of *DEFB1*, *ITIH4*, and *PADI4* were detected using RNA-seq. (D) Relative expression of the selected genes was determined using qPCR; the *GAPDH* housekeeping gene was used as a reference, and samples mock-infected with ASFV were used as negative controls. The values are means and SD. *, *P* < 0.05; **, *P* < 0.01; ***, *P* < 0.001; ns, not significant.

RNA sequencing is frequently used to screen for differentially expressed genes (DEGs) across different biological incidents. To identify the host factors that were significantly upregulated and downregulated upon infection with the deletion mutant, we performed RNA sequencing analysis using BMDMs infected with ASFV-GS and ASFV-GS-Δ18R/NL/UK. Total RNA was extracted from BMDMs infected with ASFV-GS or ASFV-GS-Δ18R/NL/UK 24 hpi. The transcriptomes were sequenced, and DEGs are shown in Fig. 5A and Tables S1 and S2. Four hundred three downregulated and 28 upregulated genes were identified in BMDMs infected with ASFV-GS-Δ18R/NL/UK. Kyoto Encyclopedia of Genes and Genomes (KEGG) analysis of the DEGs revealed that these genes were mainly clustered into several pathways, including different viral infection pathways, the RIG-I-like receptor signaling pathway, and the JAK-STAT signaling pathway (Fig. 5B). Most of these pathways are associated with viral infections and immune response.

Several genes that ranked high and low in differential expression levels were selected to determine the reliability of RNA-seq results using qPCR. The results of the analysis of high-ranking genes *H3.1* (LOC110261664; rank, 2) and *PADI4* (ENSSSCG00000003483; rank, 4) and low-ranking genes *DEFB1* (ENSSSCG00000027555; rank, 12) and *ITIH4* (ENSSSCG00000011453; rank, 37) revealed that the expression levels of these four genes in the ASFV-GS-Δ18R/NL/UK group were significantly higher than those in the ASFV-GS group (Fig. 5C and D). This finding suggests the high reliability of the RNA-seq data.

**FIG 6:**
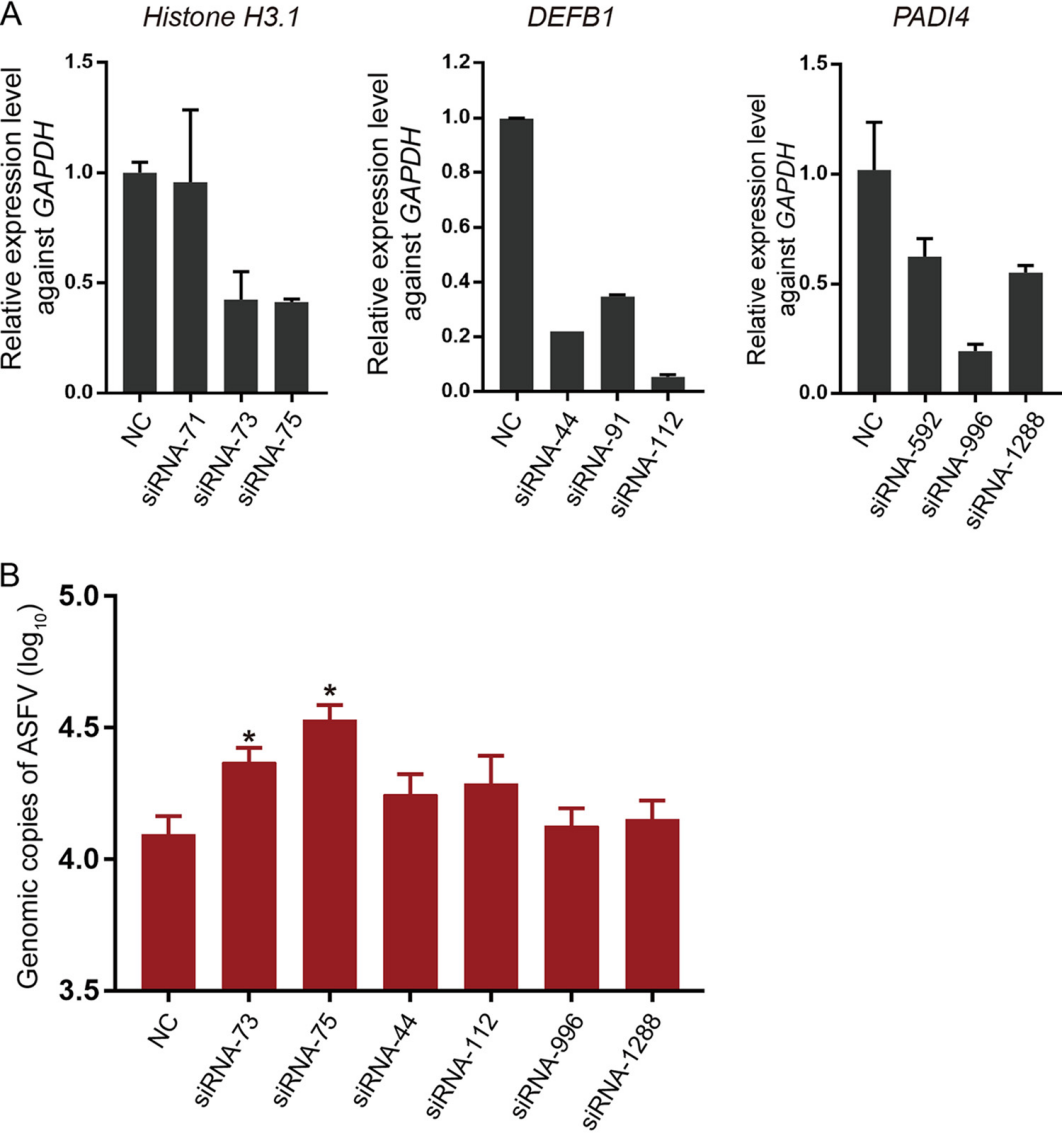
Effect of inhibition of the expression of *H3.1*, *DEFB1*, and *PADI4* on replicating ASFV. (A) Inhibitory efficiencies of different siRNAs against the corresponding genes. The swine *GAPDH* housekeeping gene was used as a reference. (B) Significant differences were detected in the replication of ASFV-GS after knocking down the host *H3.1* gene, unlike those observed upon knockdown of the other two genes. NC, nonfunctional siRNA treatment. The values are means and SD. *, *P* < 0.05.

Furthermore, to investigate the role of these genes in the replication of ASFV in BMDMs, small interfering RNAs (siRNAs) targeting *H3.1*, *DEFB1*, and *PADI4* were designed and synthesized to perform knockdown experiments. Viral genome copies were quantified using qPCR; the results showed that siRNA-75, siRNA-112, and siRNA-996, targeting *H3.1*, *DEFB1*, and *PADI4*, respectively, induced the greatest reduction in gene expression (Fig. 6A). However, downregulation of only *H3.1* induced significantly higher levels of viral replication in BMDMs at 24 hpi (*P* < 0.05), while knockdown of the other two host genes had no significant effect on ASFV replication (*P > *0.05) (Fig. 6B). These results suggest that downregulation of the histone H3.1 gene, but not that of *DEFB1* or *PADI4*, promotes the replication of ASFV *in vitro*.

### ASFV *MGF110-7L* was downregulated in the deletion mutant.

**FIG 7:**
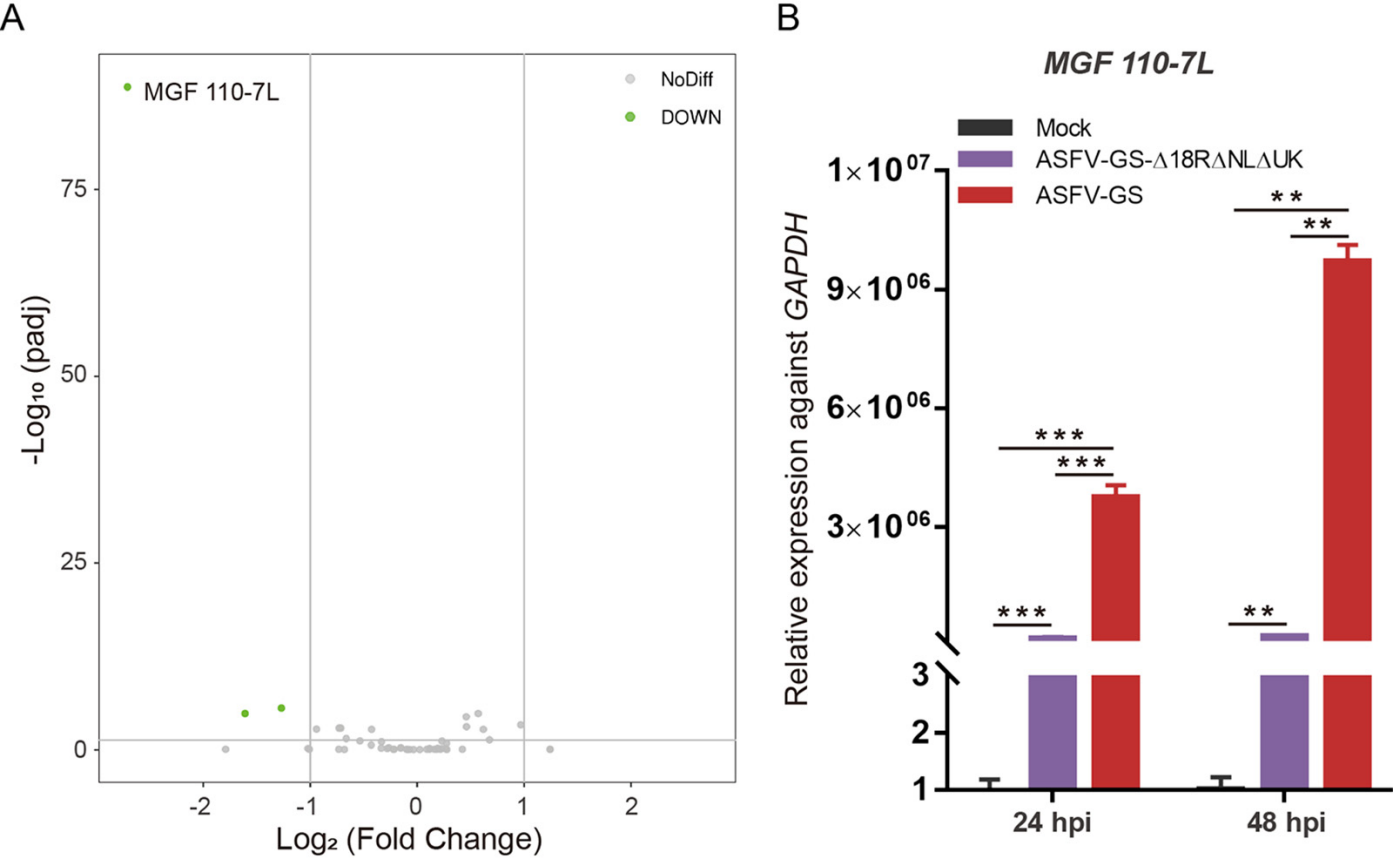
RNA sequence analysis of ASFV transcriptome. (A) Volcano plot showing the DEGs. Green plots indicate downregulated genes. (B) Relative expression determined using qPCR against *GAPDH* (host housekeeping gene) at 24 hpi and 48 hpi. Samples without ASFV infection were used as negative controls (Mock). **, *P* < 0.01; ***, *P* < 0.001.

Our results showed that targeted gene deletion in this study had no considerable effect on the neighboring genes (Fig. 2A); however, whether the expression of other viral genes was affected remains unknown. To the best of our knowledge, this issue is of little concern. Given that ASFV encodes many proteins (>150), it would be interesting and meaningful to study the transcriptome changes in these vaccine candidate strains. RNA-seq was performed on BMDMs infected with ASFV-GS or its mutant at 48 hpi. The ASFV Georgia 2007/1 genome assembly (NC_044959) isolate was used as a reference for differential expression analysis; the results showed that *MGF110-7L* of the deletion mutant was significantly downregulated compared to that in the parental virus strain (Fig. 7A). To rule out the possibility of DNA mutations affecting gene expression, the DNA sequence of *MGF110-7L* were analyzed by Sanger sequencing. The results show that no mutations occurred in the open reading frame or upstream or downstream of *MGF110-7L* gene (Fig. S2). Otherwise, we confirmed the gene expression change using qPCR and found that the ASFV *MGF110-7L* gene was significantly downregulated at both 24 and 48 hpi (Fig. 7B). These results indicate that ASFV *MGF110-7L* may be an important virulence-related gene.

## DISCUSSION

To attenuate the highly virulent ASFV-GS virus strain, we simultaneously deleted three adjacent genes from its genome. The results of immunization experiments showed that the recombinant strain was highly attenuated in pigs. No obvious fever response or reduced levels of viremia were detected in inoculated pigs. Importantly, no ASFV genome was detected in the sera of the contact animals, indicating that this recombinant virus strain may lack the ability to infect contact pigs under laboratory conditions. Additionally, the genomic manipulation and loss of these ASFV genes might not affect the multiplication of this deletion mutant *in vitro*. These two characteristics are important for an attenuated virus strain to be used as a LAV (4, 8).

According to previous reports, *DP148R* is expressed only temporarily early after ASFV infection. Deleting this gene from the ASFV isolates Benin 97/1 and Georgia 2007/1 does not affect virus replication in macrophages *in vitro* (13, 15). However, targeted deletion of this gene from genotype I ASFV (Benin 97/1) and genotype II ASFV (Georgia 2007/1 and HLJ/18) strains resulted in different effects on virulence. BeninΔDP148R was highly attenuated in pigs and provided the inoculated pigs with 100% (5/5) protection against the parental virus challenge. However, the pig immunization experiment results suggest that the virulence of GeorgiaΔDP148R and HLJ/18ΔDP148R is not distinct from that of their parental virus (15, 28). These differences are likely due to the differences in other viral proteins closely related to DP148R.

The ASFV genes *NL* and *UK* were first reported in the 1900s, and researchers studied their functions in isolate E70. Deleting either of the two genes did not affect viral growth in macrophages *in vitro*, greatly reduced virulence in pigs, and provided strong protection against the parental isolate E70 (17, 18). The authors also mentioned that the combination deletion of the two genes did not further attenuate E70. Different results were obtained by targeting the two genes in other ASFV strains. In 2013, researchers deleted *NL* and *UK* from the OUR T88/3 isolate genome, which has been attenuated naturally and could provide high-level protection against homology challenges. They found that deletion of the two genes from OUR T88/3 failed to improve the safety of this strain and reduced its ability to protect the pigs from parental virus challenge (21). Studies were also carried out on the genotype II ASFV isolate Georgia 2007/1, and ASFV-G-Δ9GL/ΔUK was produced by deleting the genes *9GL* (*B119L*) and *UK*. Results showed that the replication of this deletion mutant was significantly decreased compared to that of the parental virus and that the mutant conferred strong protection to pigs against homology challenge (12).

Additionally, knocking out *NL* from ASFV-G-Δ9GL/ΔUK further reduced virus replication in macrophages but did not induce protection against parental virus challenge (20). Given the high homology between NL and UK among different ASFV isolates, the structures and functions of viral proteins are unlikely to differ substantially. Therefore, the genetic background may be the most significant determinant of the differential results. The ASFV genome encodes more than 150 proteins. In a specific ASFV strain, proteins interacting with the given proteins are assumed to be different, which may be a vital basis for virulence. The relationship between these viral proteins is highly significant but remains less studied. Our results suggest that genetic background is a factor that must be considered when vaccine strains are developed by using gene recombination.

The results of ASFV genome manipulation were examined using qPCR and RNA-seq. As expected, the target genes (*DP148R*, *NL*, and *UK*) were functionally lost, and the neighboring genes were unaffected by molecular events. Interestingly, we found that the ASFV gene *MGF110-7L* was downregulated significantly in the transcriptome of ASFV-GS-Δ18R/NL/UK but not in that of ASFV-GS. ASFV MGF110-7L has been reported to trigger stress granule (SG) formation and inhibit host cell translation by inducing phosphorylation of the α subunit of eukaryotic initiation factor 2 (eIF2α) (29). This process may play an important role in the virulence of ASFV in pigs. Further studies are needed to determine whether *MGF110-7L* has other functions. The relationship between the downregulation of *MGF110-7L* and three deleted genes remains unclear. Given that *MGF110-7L* and *DP71L* are found to be functionally linked to eIF2α (29, 30), it is reasonable to speculate that altering one gene may have an impact on the other. In addition, these genes might have other, as-yet-unidentified, functions. More research is required to determine the precise mechanism underlying the downregulation of *MGF110-7L* and the deletion of these three genes.

Identifying differentially expressed genes in BMDM cultures infected with the attenuated virus strain would be valuable for antiviral drug development (7). Therefore, we performed RNA-seq using BMDMs infected with the viral deletion mutant or with its parental strain. The 28 upregulated and 403 downregulated genes identified in this study may be valuable resources for anti-ASFV drug development. For example, histone H3.1 and its variant H3.3 are highly associated with herpes simplex virus 1 (HSV-1) genome replication, and incorporating the H3.3 gene into the HSV-1 genome facilitates its transcription (31, 32). Similarly, defensin β1, encoded by *DEFB1*, is expressed by monocytes, macrophages, and certain dendritic cells (DCs) and has antiviral effects in humans (33, 34); peptidyl arginine deiminase 4, encoded by *PADI4*, plays an important role in inflammation and immune response (35); and the plasma protein inter-alpha-trypsin inhibitor heavy chain H4, encoded by *ITIH4*, is abundant in the sera of survivors of severe acute respiratory syndrome coronavirus 2 (SARS-CoV-2) infection (36) and is involved in the acute-phase response to virus infection or inflammation (37, 38). Based on these previous reports, we speculated that these genes might function in the attenuation of ASFV-GS-Δ18R/NL/UK in pigs.

Furthermore, RNA interference (RNAi) was performed to determine the functions of *H3.1*, *DEFB1*, and *PADI4* during the replication of the ASFV genome. qPCR results revealed that the knockdown of *DEFB1* and *PADI4* in BMDM cultures only slightly promoted ASFV genome replication; however, the differences were not significant. This finding may be explained by the expression pattern of defensin β1, encoded by *DEFB1*, which is often induced by a viral infection, unlike α defensins, which are expressed constitutively (39) and always function as chemokines to trigger immune responses (40). This indirect antiviral effect often occurs in organisms or organs and is difficult to detect in primary macrophage cultures *in vitro*. *H3.1* downregulation leads to significantly increased levels of ASFV-GS replication. Cellular DNA in the nucleus is associated with histones composed of H2A, H2B, H3, H4, and other proteins to form chromatin. The chromatin structure controls the access of transcription factors to DNA and has been demonstrated to be important in regulating gene expression, DNA replication, and DNA repair (41). Previous studies have shown that the genomes of several DNA viruses, such as HSVs and papillomaviruses, are achromatized during their life cycle, causing histones to be associated with the regulation of viral transcription and replication (42–44). The association of histones with the transcription and replication of viral genomes has been demonstrated in various studies (45–47). However, the exact mechanisms underlying the action of these histones remain unknown. The genome of ASFV is as large as 170 to 190 kb, which is markedly larger than the genomes of papillomaviruses (only approximately 8 kb). Therefore, regulatory pathways associated with histones are likely to function in the replication of ASFV. More studies are needed to investigate the roles of *H3.1* and other DEGs identified herein during immunoprotection *in vivo*.

We constructed an attenuated ASF virus strain by deleting the *DP148R*, *NL*, and *UK* viral genes. Our results showed that this strain was highly attenuated and conferred full protection against homologous challenges. Various DEGs were identified in the BMDMs infected with the recombinant virus strain. ASFV *MGF110-7L* was significantly downregulated in ASFV-GS-Δ18R/NL/UK. Our study may provide valuable targets for vaccine development and antiviral treatments.

## MATERIALS AND METHODS

### Ethics and biosafety.

All animal experiments in this study were performed in accordance with the recommendations in the Guide for the Care and Use of Laboratory Animals of the Ministry of Science and Technology of the People’s Republic of China. The protocols were approved by the Committee on the Ethics of Animal Experiments of the Lanzhou Veterinary Research Institute, Chinese Academy of Agricultural Science (CAAS). All live viruses were manipulated within the biosafety level 3 (BSL-3) facilities at the Lanzhou Veterinary Research Institute of CAAS approved by the Ministry of Agriculture and Rural Affairs and the China National Accreditation Service for Conformity Assessment.

### Primary cell cultures.

Primary swine macrophage cultures were derived from swine bones, as previously described (17). Briefly, 1-month-old pigs were sacrificed to obtain the thigh bones. Macrophage fractions were removed and cultured in uncoated petri dishes containing medium composed of RPMI 1640 medium with 10% fetal bovine serum (FBS), 40 ng/mL macrophage colony-stimulating factor (M-CSF), and penicillin-streptomycin (1×) for at least 5 days at 38.5°C and 5% CO_2_. Adherent cells were detached from the plastic culture dishes using a cell scraper and stored in liquid nitrogen at a density of 10^7^ per mL.

### Construction and analysis of recombinant ASFV-GS-Δ18R/NL/UK.

Mutant ASFVs were constructed by homologous recombination between the ASFV-GS isolate genome and transfer vectors in swine BMDM cultures. First, the donor vector, p72GFP-NL/UK, containing a reporter gene cassette (reporter enhanced GFP [EGFP] gene promoted by the ASFV p72 gene promoter) and a left and right homologous recombinant arm, was constructed and sequenced. This construction replaced a 602-nucleotide segment (184068 to 184669, covering the viral MGF 360-18R, UK, and NL genes) with a GFP reporter cassette. Second, BMDM cultures were infected with ASFV-GS and transfected with p72GFP-NL/UK. Recombinant viruses could be identified by EGFP signals under a fluorescence microscope and purified by successive rounds of EGFP-positive single-cell sorting. The purity of ASFV-GS-Δ18R/NL/UK was assessed using PCR. The internal reference p72 open reading frame (ORF) and the gene deletion were detected by using the primers listed in Table 1. The products of these primers were 120 bp and 400 bp.

**TABLE 1:** Primers used in this study

Purpose	Name	Sequence (5′-3′)
Purity test of ASFV-GS-Δ18R/NL/UK	181UK-F	AAACTACGCTCGCAGCGCAAAAAG
	181UK-R	ATCCACGACAACTGATTTCTCAGA
	p72-F	CCGGGGTATTCGCAGTAGTA
	P72-R	GCAGCTTCAAACGTTTCCTC
qPCR analysis	qH3.1-F	ACTTCAAGACGGACCTGCGCTT
	qH3.1-R	TTGGGCATGATGGTGACGCGCT
	qDEFB1-F	TGCTGACTGTCTGCCTCCTC
	qDEFB1-R	AATCCTGTTGAAGAGCGGGC
	qITIH4-F	TTGCTGCTGGCTGTCCTTCA
	qITIH4-R	TCCTGCACAGCACTGCCCTT
	qPADI4-F	AGACTCAGCTGGATGTCTAC
	qPADI4-R	AGGGACCTGTGCGATTCTTT
	qMGF110-7L-F	ATGCTGGTGATTATCCTGGGAA
	qMGF110-7L-R	TAAGTGCACCAGTATCCAAGTT
	qL11L-F	AGGATGTTGGAGCCAATGTTAG
	qL11L-R	AGAGTGCAATTGTTATAAGTTT
	qMGF360-18R-F	AGCCTCAAGGGACTAGTCTCCG
	qMGF360-18R-R	CTACTGGAGCAGCAGTAAGAAG
	qDP71L-F	ATGTCCATTTTGCCACAGCC
	qDP71L-R	ATAGGAGCTCCTCCACGCTC
	qDP96R-F	ATGTCTACACATGATTGTTCTC
	qDP96R-R	TCAGGTATATGATTCGCTCCAG
	qMGF360-21R-F	ATGTCTACTCCACTTTCTCTAC
	qMGF360-21R-R	AAATCGTAATCGGCGCTTCATG
	qP72-F	CCGGGTACAATGGGTCTTCC
	qP72-R	CGCAACGGATATGACTGGGA
	qGAPDH-F	TCGGAGTGAACGGATTTGGC
	qGAPDH-R	TGCCGTGGGTGGAATCATAC

### Growth curve of ASFV-GS and ASFV-GS-Δ18RΔNLΔUK.

The growth characteristics of ASFV-GS-Δ18R/NL/UK viruses were evaluated in BMDM cultures. BMDMs were prepared in 96-well plates and infected at a multiplicity of infection (MOI) of 0.01 (based on the previously determined HAD_50_). After 1 h of adsorption at 37°C, the supernatant was removed, and the cells were rinsed twice with phosphate-buffered saline (PBS). Fresh macrophage medium was added, and cells were incubated for 2, 24, 48, 72, and 96 h at 37°C under 5% CO_2_. Cells were frozen (−80°C) and thawed twice to collect virus samples at appropriate times. ASFV-GS was kindly provided by Keshan Zhang of the Lanzhou Veterinary Research Institute, Chinese Academy of Agricultural Sciences.

### Virus titration.

Titers of ASFV-GS and ASFV-GS-Δ18R/NL/UK were determined using the HAD_50_ test in BMDM cultures. The presence of virus was tested by hemadsorption (HA) in 96-well plates, and virus titers were calculated using the method of Reed and Muench (48, 49).

### Animal experiments.

The virulence of ASFV-GS-Δ18R/NL/UK was assessed relative to that of the parental ASFV-GS using mature commercial-breed swine. Groups of pigs (*n* = 5) were inoculated i.m. with 10^4^ HAD_50_ of ASFV-GS-Δ18R/NL/UK or ASFV-GS. In the protection experiments, animals were challenged i.m. with 10^2^ HAD_50_ of the parental virulent ASFV strain at 19 dpi. Clinical signs (anorexia, depression, fever, purple skin discoloration, staggering gait, diarrhea, and cough) and body temperature were recorded daily throughout the experiment. Sera, oral swabs, nasal swabs, and anal swabs from experiment animals were collected every other day. The serum was isolated from the blood samples which were collected from the jugular vein.

### Detection of ASFV p30-specific antibody with a Block ELISA kit.

ASFV p30 (CP204L) antibody was examined by using a Block ELISA kit (Lanzhou Shouyan Biotechnology Co., Ltd.; lot glwe-sj). Briefly, viral antigens were produced from Escherichia coli. ASFV p30-specific antibodies were analyzed using an anti-swine IgG–horseradish peroxidase conjugate and peroxidase substrate. The ELISA plates were read at an optical density of 450 nm (OD_450_) using a Multiskan FC microplate photometer (Thermo Fisher). The blocking rates were calculated with help of a standard curve established independently in each assay.

### RNA-seq.

Total RNA was extracted from BMDM cultures infected with ASFV CN/GS 2018 or ASFV-GS-Δ18R/NL/UK using TRIzol reagent (Invitrogen). Thereafter, quantification and qualification were performed. Three micrograms of RNA per sample was used for library preparation using the NEBNext Ultra RNA library preparation kit for Illumina (New England Biolabs [NEB]), according to the manufacturer’s recommendations. The libraries were sequenced on an Illumina HiSeq platform, and 150-bp paired-end reads were generated. Differential expression analysis was performed using the DESeq2 R package (version 1.16.1). The resulting *P* values were adjusted using Benjamini and Hochberg’s approach for controlling the false discovery rate. Genes with an adjusted *P* value of <0.05, found by DESeq2, were determined to be differentially expressed. Gene Ontology (GO) enrichment analysis of DEGs was performed using the topGO R package, where gene length bias was corrected. GO terms with *P* values of <0.05 were considered significantly enriched by DEGs. The ClusterProfiler R package was used to test the statistical enrichment of DEGs in the KEGG pathways.

### qPCR.

For ASFV genome copy number determination, qPCR was carried out as recommended by the OIE 2019 terrestrial animal health code (50, 51). Briefly, viral DNA was extracted using the QIAamp all-nucleic-acid MDx kit (Qiagen) according to the manufacturer’s recommendations. A TaqMan probe was designed based on an alignment of 54 available ASFV sequences for the 3′ end of p72. The sensitivity of the qPCR method we used to determine ASFV genome copies is >2 copies/μL. This translates to a lower limit of 10^2.305^ ASFV genome copies/100 μL. Values below this indicate the presence of viral genome, but the exact number cannot be determined.

To determine the gene expression levels, total RNA was extracted from relevant cells using the TRIzol reagent (Invitrogen), according to the manufacturer’s instructions. Reverse transcription reactions for mRNAs were performed using the PrimeScript RT reagent kit (TaKaRa). To determine relative mRNA abundance, quantitative PCR was performed with Powerup SYBR green master mix (Applied Biosystems) on a StepOnePlus system (ABI); the primers are listed in Table 1. Data were analyzed using the StepOnePlus software. The *GAPDH* expression level was used for normalization.

### Statistical analysis.

All experiments were repeated independently at least three times. Statistical analyses were performed using GraphPad Prism 5.0 and an unpaired, two-tailed Student’s *t* test. *P* values of <0.05 were considered statistically significant.
