# Supplementary material for: Deletion of DP148R, DP71L, and DP96R Attenuates African Swine Fever Virus, and the Mutant Strain Confers Complete Protection against Homologous Challenges in Pigs
Source: J Virol. 2023 Apr 5;97(4):e00247-23. doi: 10.1128/jvi.00247-23 (PMC10134827; doi:10.1128/jvi.00247-23)
Supplement: Supplemental file 3 — Fig. S1 and S2. Download jvi.00247-23-s0003.pdf, PDF file, 6.0 MB [file jvi.00247-23-s0003.pdf]

Figure S1

A

```
                  **         *   *  *
Georiga 2007/1   MGR-RRKKRTNDAKHVHFATAVEVWEADDIERKGPWEQVAVDRFRFQRRI
Czech            MGR-RRKKRTNDAKHVHFATAVEVWEADDIERKGPWEQVAVDRFRFQRRI
Belgium 2018/1   MGR-RRKKRTNDAKHVHFATAVEVWEADDIERKGPWEQVAVDRFRFQRRI
HLJ/2018         MGR-RRKKRTNDAKHVHFATAVEVWEADDIERKGPWEQVAVDRFRFQRRI
CN/GS/2018       MGR-RRKKRTNDAKHVHFATAVEVWEADDIERKGPWEQVAVDRFRFQRRI
OURT_88/3        MGGRRRKKRTNDTKHVRFAAAVEVWEADDIERKGPWEQVAVDRFRFQRRI
BA71V            MGGRRRKKRTNDTKHVRFAAAVEVWEADDIERKGPWEQVAVDRFRFQRRI

Georiga 2007/1   ASVEELSAVLLRQKKLLEQQ
Czech            ASVEELSAVLLRQKKLLEQQ
Belgium 2018/1   ASVEELSAVLLRQKKLLEQQ
HLJ/2018         ASVEELSAVLLRQKKLLEQQ
CN/GS/2018       ASVEELSAVLLRQKKLLEQQ
OURT_88/3        ASVEELSTVLLRQKKLLEQQ
BA71V            ASVEELSTVLLRQKKLLEQQ
```

B

```
                       **         *  **                  *
Georiga 2007/1   MSTHDCSLKEKPVDNDISEKSVVVDNAPEKPAGANHIPEKSAREMTSSEW
Czech            MSTHDCSLKEKPVDNDISEKSVVVDNAPEKPAGANHIPEKSAREMTSSEW
Belgium 2018/1   MSTHDCSLKEKPVDNDISEKSVVVDNAPEKPAGANHIPEKSAREMTSSEW
HLJ/2018         MSTHDCSLKEKPVDNDISEKSVVVDNAPEKPAGANHIPEKSAREMTSSEW
CN/GS/2018       MSTHDCSLKEKPVDNDISEKSVVVDNAPEKPAGANHIPEKSAREMTSSEW
OURT_88/3        MSTHDCFSKEKPVDNDIFEKFSVVDNAPEKPAGANHIPEKFAREMTSSEW
BA71V            MSTHDCFSKEKPVDNDIFEKFSVVDNAPEKPAGANHIPEKFAREMTSSEW

                                                     *
Georiga 2007/1   IAEYWKGIKRNDVPCCCPRKMTSADKKFSVFGKGSLMRSIQKNN
Czech            IAEYWKGIKRNDVPCCCPRKMTSADKKFSVFGKGSLMRSIQKNN
Belgium 2018/1   IAEYWKGIKRNDVPCCCPRKMTSADKKFSVFGKGSLMRSIQKNN
HLJ/2018         IAEYWKGIKRNDVPCCCPRKMTSADKKFSVFGKGSLMRSIQKNN
CN/GS/2018       IAEYWKGIKRNDVPCCCPRKMTSADKKFSVFGKGSLMRSIQKNN
OURT_88/3        IAEYWKGIKRNDVPCCCPRKMTSADKKFSVFGKGSLI RSIQKNN
BA71V            IAEYWKGIKRNDVPCCCPRKMTSADKKFSVFGKGSLI RSIQKNN
```

Figure S2

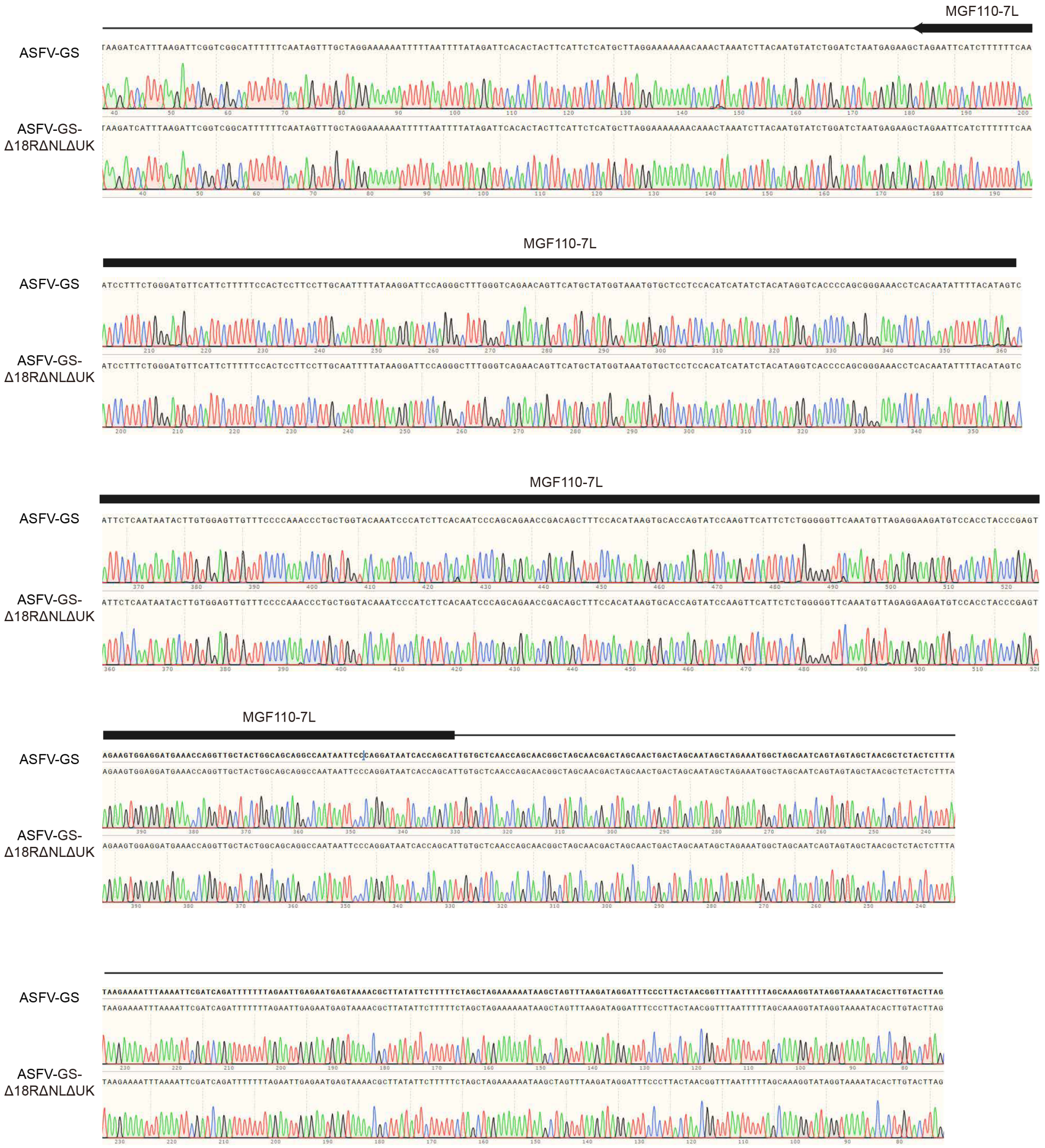
MGF110-7L
ASFV-GS
ASFV-GS-Δ18RΔNLΔUK
MGF110-7L
ASFV-GS
ASFV-GS-Δ18RΔNLΔUK
MGF110-7L
ASFV-GS
ASFV-GS-Δ18RΔNLΔUK
MGF110-7L
ASFV-GS
ASFV-GS-Δ18RΔNLΔUK
ASFV-GS
ASFV-GS-Δ18RΔNLΔUK

Figure legends

Fig. S1. Protein sequence similarity analysis of NL and UK among different ASFV isolates. A. Comparison of the sequences among the indicated ASFV isolate of the NL viral protein. B. Sequence alignment of the indicated strains of the UK. Different amino acid residues among strains are marked by asterisks.

Fig. S2. DNA sequencing of ASFV MGF110-7L by Sanger-seq. Sequences of viral DNA region covering open reading frame, upstream (> 270 bp) and downstream (> 150 bp) of MGF110-7L were analyed, and no variations been found between ASFV-GS and ASFV-GS-Δ18R/NL/UK.
